# A putative relay circuit providing low-threshold mechanoreceptive input to lamina I projection neurons via vertical cells in lamina II of the rat dorsal horn

**DOI:** 10.1186/1744-8069-10-3

**Published:** 2014-01-17

**Authors:** Toshiharu Yasaka, Sheena YX Tiong, Erika Polgár, Masahiko Watanabe, Eiichi Kumamoto, John S Riddell, Andrew J Todd

**Affiliations:** 1 Institute of Neuroscience and Psychology, College of Medical, Veterinary and Life Sciences, University of Glasgow, Glasgow G12 8QQ, UK; 2 Department of Anatomy and Physiology, Faculty of Medicine, Saga University, Saga, Japan; 3 Department of Anatomy, Hokkaido University School of Medicine, Sapporo 060-8638, Japan

## Abstract

**Background:**

Lamina I projection neurons respond to painful stimuli, and some are also activated by touch or hair movement. Neuropathic pain resulting from peripheral nerve damage is often associated with tactile allodynia (touch-evoked pain), and this may result from increased responsiveness of lamina I projection neurons to non-noxious mechanical stimuli. It is thought that polysynaptic pathways involving excitatory interneurons can transmit tactile inputs to lamina I projection neurons, but that these are normally suppressed by inhibitory interneurons. Vertical cells in lamina II provide a potential route through which tactile stimuli can activate lamina I projection neurons, since their dendrites extend into the region where tactile afferents terminate, while their axons can innervate the projection cells. The aim of this study was to determine whether vertical cell dendrites were contacted by the central terminals of low-threshold mechanoreceptive primary afferents.

**Results:**

We initially demonstrated contacts between dendritic spines of vertical cells that had been recorded in spinal cord slices and axonal boutons containing the vesicular glutamate transporter 1 (VGLUT1), which is expressed by myelinated low-threshold mechanoreceptive afferents. To confirm that the VGLUT1 boutons included primary afferents, we then examined vertical cells recorded in rats that had received injections of cholera toxin B subunit (CTb) into the sciatic nerve. We found that over half of the VGLUT1 boutons contacting the vertical cells were CTb-immunoreactive, indicating that they were of primary afferent origin.

**Conclusions:**

These results show that vertical cell dendritic spines are frequently contacted by the central terminals of myelinated low-threshold mechanoreceptive afferents. Since dendritic spines are associated with excitatory synapses, it is likely that most of these contacts were synaptic. Vertical cells in lamina II are therefore a potential route through which tactile afferents can activate lamina I projection neurons, and this pathway could play a role in tactile allodynia.

## Background

Lamina II of the spinal dorsal horn contains numerous densely packed neurons, which have axons that arborise locally and remain within the spinal cord [1,2]. Between a quarter and a third of these cells are GABAergic/glycinergic inhibitory interneurons [3,4], while the remainder are excitatory, glutamatergic interneurons [5-7]. Lamina II interneurons are diverse, and numerous attempts have been made to classify them into functional populations, based on morphological, electrophysiological or neurochemical criteria [2,5,6,8-14]. Among the excitatory interneurons, one class that has been recognised in several studies consists of vertical cells, which usually have their cell body in the outer part of the lamina (IIo) and cone-shaped dendritic trees that extend in a ventral direction [5,6,9,11,13,15-18]. Many vertical cells have numerous spines, or stalk-like appendages, and these were previously known as stalked cells in studies of the cat spinal cord and spinal trigeminal nucleus [19-21].

Lamina I projection neurons represent a major output from the superficial dorsal horn. They have axons that cross the midline and pass through the contralateral ventral quadrant, constituting a significant part of the ascending anterolateral tract (ALT). They project to a variety of brainstem structures, including the lateral parabrachial area (LPb), periaqueductal grey matter, nucleus of the solitary tract and thalamus [2,22,23]. We have shown that the vast majority of lamina I projection neurons in the rat lumbar enlargement can be retrogradely labelled from the LPb [23-25], and the electrophysiological properties of lamina I spinoparabrachial neurons [26-29] are therefore likely to reflect those of all ALT cells in this lamina. Recordings from these cells in anaesthetised rats indicate that virtually all (96–100%) respond to noxious stimuli, with a few also being activated by innocuous mechanical stimulation [26,28]. Some lamina I projection neurons also respond to pruritic stimuli, and are thus likely to convey information perceived as itch [30].

Primary afferent input to the dorsal horn is arranged in a highly organised way, with nociceptive and thermoreceptive afferents terminating mainly in laminae I and IIo, while low-threshold mechanoreceptive (LTMR) inputs arborise in a region extending ventrally from the inner half of lamina II (IIi) [2]. It has been reported that the proportion of lamina I projection neurons that respond to low-threshold mechanical stimuli increases following nerve injury [29], and this is thought to contribute to the tactile allodynia seen in neuropathic pain. Following blockade of spinal inhibitory transmission there is an increased input from large myelinated (Aβ) afferents (presumed LTMRs) to lamina I neurons, and it has been suggested that this is conveyed through polysynaptic pathways involving excitatory interneurons [31,32].

Vertical cells in lamina II could potentially provide a route through which myelinated LTMR (A-LTMR) primary afferents activate lamina I projection neurons, since their dendrites often extend into the region where these afferents terminate, and their axons frequently arborise in lamina I [5,6,9,17,19,20]. The aim of this study was therefore to determine whether vertical cells receive contacts from boutons belonging to A-LTMRs. Since many of these afferents express the vesicular glutamate transporter VGLUT1, and these are the main source of VGLUT1-immunoreactive terminals in this region [33], we initially looked for contacts between VGLUT1^+^ boutons and vertical cell dendrites. However, since not all VGLUT1-immunoreactive boutons are of primary afferent origin [34], we also examined three vertical cells that were identified in rats that had received an injection of cholera toxin B subunit (CTb) into the sciatic nerve, in order to bulk label A-LTMRs.

## Methods

All animal experiments were approved by the Ethical Review Process Applications Panel of the University of Glasgow or the Saga University Animal Care and Use Committee. They were performed in accordance with the European Community directive 86/609/EC, the UK Animals (Scientific Procedures) Act 1986 and the “Guiding Principles for the Care and Use of Animals in the Field of Physiological Science” of the Physiological Society of Japan.

### VGLUT1 contacts on vertical cells

Seven of the glutamatergic vertical cells that were identified in our previous study [6] were tested for the presence of contacts from VGLUT1-immunoreactive boutons. The cells had been recorded with the blind whole-cell patch-clamp method in sagittal spinal cord slices taken from young adult (6–10 week old) Wistar rats, using Neurobiotin-filled pipettes, as described previously [6]. A single 60 μm thick section that had been reacted with avidin conjugated to Rhodamine Red (1:1000; Jackson Immunoresearch, West Grove, PA, USA) and contained part of the dendritic tree of the recorded cell was taken from each of these slices. This was incubated free-floating in goat antibody against VGLUT1 [35] (1:500), followed by species-specific donkey anti-goat IgG conjugated to Alexa 488 or Alexa 555 (Life Technologies, Paisley, UK; 1:500) or DyLight 649 (Jackson Immunoresearch; 1:500). Sections were scanned with a Zeiss LSM 710 confocal microscope (with Argon multi-line, 405 nm diode, 561 nm solid state and 633 nm HeNe lasers) through a 63× oil-immersion lens (NA 1.4) with the pinhole set to 1 Airy unit, to create image stacks (0.3 or 0.5 μm z-separation) of those parts of the dendritic trees that lay within the plexus of VGLUT1-immunoreactive axons. These image stacks were analysed with Neurolucida for Confocal software (MBF Bioscience; Williston, VT, USA).

The dorsal limit of the dense plexus of VGLUT1 staining, which occupies laminae IIi-VI [33,36], was initially drawn, and then the VGLUT1 channel was hidden. All dendritic spines belonging to the recorded cells that lay below this limit (i.e. within the VGLUT1 plexus) were identified. The VGLUT1 channel was then switched on, and any VGLUT1-immunoreactive boutons that contacted the spines were recorded.

### VGLUT1 contacts on vertical cells in rats that had received sciatic nerve injections

The methods used for injection of CTb into the sciatic nerve, and for obtaining spinal cord slices from adult rats were similar to those described previously [6,37,38]. Briefly, 2 male Wistar rats (7 weeks old) were deeply anaesthetized with isoflurane. The left sciatic nerve was exposed and injected with 5 μl of 1% CTb (Sigma-Aldrich, St Louis, MO, USA). Four days later, the animals were deeply anaesthetized with isoflurane. After thoracolumbar laminectomy, the spinal cord was removed into ice-cold dissection solution (mM: NaCl 0, KCl 1.8, KH2PO4 1.2, CaCl2 0.5, MgCl2 7, NaHCO3 26, glucose 15, sucrose 254, oxygenated with 95% O2, 5% CO2). The rats were then killed by anaesthetic overdose and decapitation. All dorsal and ventral roots were removed. The spinal cord was then glued onto an agar block and cut into 500 μm thick parasagittal slices with a microslicer (DTK-1000; Dosaka EM Co., Ltd., Kyoto, Japan). From each rat, a slice that included the sciatic nerve territory of the L4 and L5 segments was selected and transferred to a recording chamber where it was perfused with normal Krebs’ solution (identical to the dissection solution except for (mM): NaCl 127, CaCl2 2.4, MgCl2 1.3 and sucrose 0) at 10 ml min^-1^ at room temperature. Slices were perfused for at least 30 min before recording. Lamina II was identified as a translucent band across the dorsal horn under a dissecting microscope. Blind whole-cell voltage- or current-clamp recordings were made from neurons in this region as previously described [6], by using glass pipettes (7–12 MΩ) filled with a solution containing the following (mM): potassium gluconate 120, KCl 20, MgCl2 2, Na2ATP 2, NaGTP 0.5, Hepes 20, EGTA 0.5, and 0.2% Neurobiotin (pH 7.28 adjusted with KOH). Signals were acquired with a patch-clamp amplifier (Axopatch 200B, Molecular Devices, Sunnyvale, CA) and acquisition software (pCLAMP 10, Molecular Devices). Signals were lowpass filtered at 5 kHz, amplified 10-fold in voltage-clamp mode or 50-fold in current-clamp mode, sampled at 10 kHz and analysed offline using pCLAMP 9 or 10. No correction was made for the liquid junction potential.

The resting membrane potential was determined immediately after establishing the whole-cell configuration. Neurons that had a resting membrane potential less negative than -40 mV were not used for electrophysiological recording. The built-in pCLAMP membrane test was used to monitor membrane properties during recording. The protocol used to test firing patterns in this study was based on that described by Sandkühler and co-workers [13,39,40]. In our previous study, we found that all excitatory vertical cells showed firing patterns associated with A-type potassium (*I*_A_) currents (delayed or reluctant firing) [6,41]. These firing patterns depend on the holding potential, because removing inactivation of A-type potassium channels requires a hyperpolarized membrane potential. To optimise detection of *I*_A_-related firing patterns, we used a standardised protocol that involved testing each cell from three different potentials (one from between -50 and -65 mV, one from between -65 and -80 mV and one from a potential more negative than -80 mV). If an *I*_A_-related firing pattern was observed, however, the remaining firing patterns from more negative membrane potentials were not assessed. A voltage step protocol was used to assess the presence of *I*_A_, hyperpolarisation-activated currents (H-currents, *I*_h_), and currents through low threshold calcium channels (*I*_Ca_). The membrane potential was held at -50 mV (or in some cases at -40 or -30 mV) and increasing negative voltage steps of 1 s duration were applied (usually over the range -60 to -140 mV, with 10 mV steps).

The slices from these rats were initially incubated in avidin conjugated to Alexa 488 (Life Technologies; 1:500). They were then cut into 60 μm thick sections with a vibrating microtome, and these were scanned to reveal the morphology of the recorded cells. Three of these were classified as vertical cells, and sections that contained most of the dendritic trees of these cells were incubated for 3 days in a cocktail consisting of guinea pig or rabbit antibody against VGLUT1 (Millipore; 1:5,000 and Synaptic Systems, Göttingen, Germany; 1:5,000, respectively) and goat anti-CTb (List Biological Laboratories, Campbell, CA, USA; 1:500). For one of the cells, several boutons belonging to its axon were present in the section, and for this section guinea pig anti-VGLUT2 (Millipore; 1:5,000) was also included in the primary antibody cocktail. The sections were incubated overnight in species-specific fluorescent secondary antibodies (Jackson Immunoresearch) conjugated to Rhodamine Red, Pacific Blue (both 1:100) or DyLight 649 (1:500). The sections were scanned and analysed in a similar way to that described above, except that in this case, VGLUT1 boutons that contacted dendritic spines of the cells were tested for the presence of CTb-immunoreactivity. For the cell with labelled axonal boutons, we examined these for the presence of VGLUT2-immunoreactivity, which can be used to confirm that the cell is glutamatergic [5,6,33,42].

### Characterisation of antibodies

The goat and rabbit VGLUT1 antibodies were raised against the C terminal amino acid sequence (531–560, 456–561, respectively) of the rat protein, and both show a band of the appropriate size on Western blots [35,43]. The guinea pig anti-VGLUT1 antibody was raised against a 19 amino acid sequence from the rat protein and stains identical structures to the rabbit anti-VGLUT1 [33]. The anti-CTb antibody was raised against the purified protein and specificity was demonstrated by the lack of staining in regions that did not contain transported CTb. The guinea pig VGLUT2 antibody was raised against an 18 amino acid sequence from rat VGLUT2 and stains identical structures to a well-characterised rabbit antibody against VGLUT2 [33].

## Results

### Contacts from VGLUT1 boutons onto vertical cells

**Figure 1:**
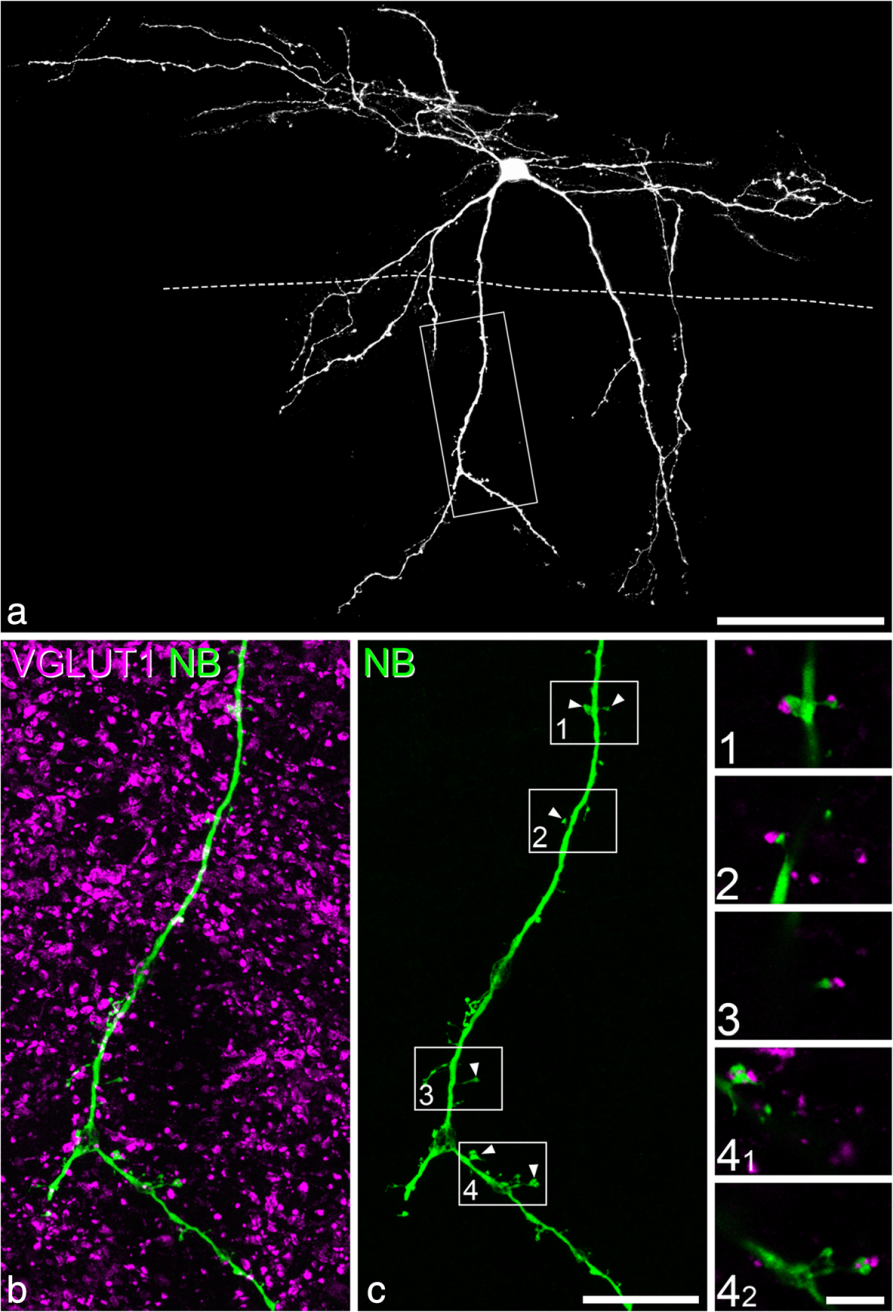
**VGLUT1 boutons contact spines belonging to a vertical cell. a**, Projected confocal image of one of the vertical cells from the study by Yasaka et al. [6]. The dashed line represents the dorsal limit of the dense plexus of VGLUT1-immunoreactive boutons that extends ventrally from lamina IIi. The box shows the area illustrated in **b** and **c**. **b**, **c** show part of a ventral dendrite of this cell scanned to reveal Neurobiotin (green) and VGLUT1 (magenta) in a projection of 62 z-sections at 0.3 μm z-spacing. The insets are from single optical sections, and show contacts between VGLUT1 boutons and individual dendritic spines at higher magnification. They correspond to the numbered boxed areas in **c**, and arrowheads show the locations of the spines that receive these contacts. Scale bars: 100 μm **(a)**, 20 μm **(b,c)**, 5 μm (insets).

We initially examined sections through 7 of the vertical cells that had been recorded in our previous study [6]. All of these cells belonged to the group that had VGLUT2-immunoreactive axons, and five of them are illustrated in Figure three of Yasaka et al. [6] (see Table 1). When sections from the slices containing these cells were incubated in antibody against VGLUT1, a dense plexus of immunoreactive axonal boutons was seen extending ventrally from lamina IIi, as reported previously [33,36,44-49]. The density of VGLUT1^+^ boutons was much lower in laminae I and IIo. In each case dendrites belonging to the cell entered this plexus, and all 7 cells received numerous contacts from VGLUT1-immunoreactive boutons, on both their dendritic shafts and spines (Figure 1). Between 55 and 454 spines were identified on the dendrites of these cells as they lay within the VGLUT1 plexus, and between 21–62% (mean 36%) of these spines received contacts from VGLUT1-immunoreactive boutons (Table 1).

**Table 1:** Contacts from VGLUT1 boutons onto the dendritic spines of 7 vertical cells

**Cell**	**Number of spines in VGLUT1 plexus**	**% spines with VGLUT1 contact**	**Cell identity in Figure three of Yasaka et al. (2010)**[6]
1	55	49	a
2	89	21	c
3	209	29	d
4	97	62	e
5	281	31	f
6	454	22	*
7	115	41	*
8	199	35	
9	97	35	
10	283	32	

* indicates cells that were not illustrated in Figure three of Yasaka et al. 2010 [6].

### Vertical cells in rats that received sciatic injections of CTb

**Figure 2:**
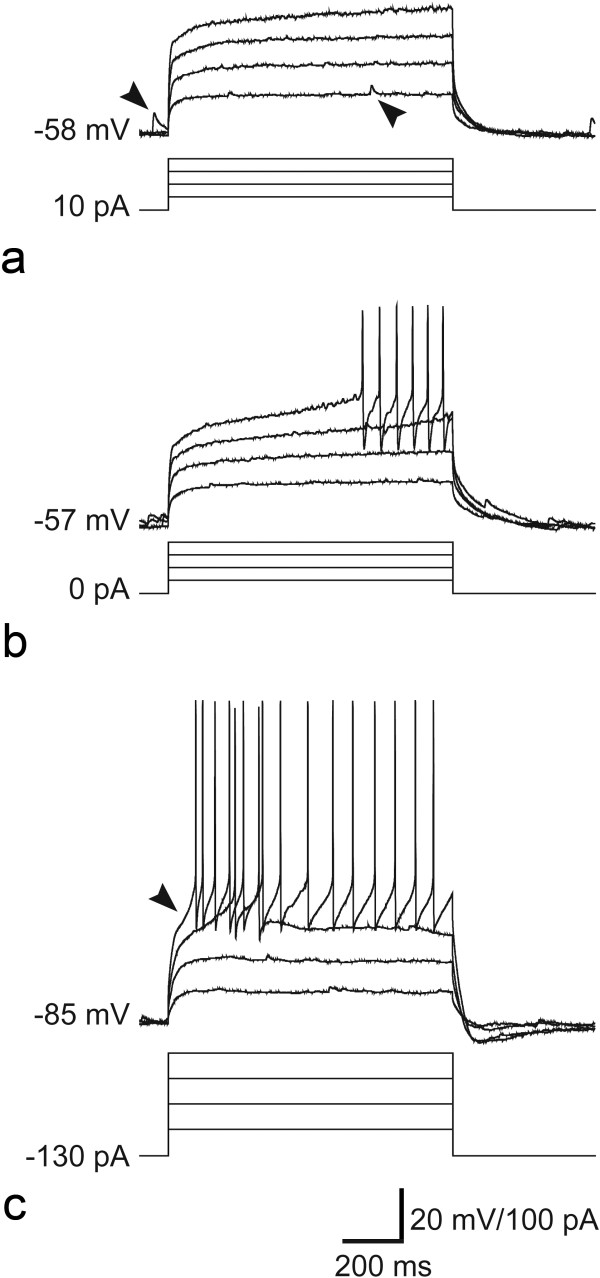
**Firing patterns of the vertical cells from the CTb injected animals.** In each case, the lower traces indicate square wave depolarising current pulses and the upper traces show the response of the cell. Figures to the left show the initial membrane voltage and current before the application of the pulses. **a**, shows traces from the neuron defined as reluctant (cell 8 in Table 1). Note that although the cell does not fire action potentials in response to depolarising current injection, it does show spontaneous EPSPs (two indicated with arrowheads). **b**, **c**, show traces from the two neurons with delayed firing patterns (cells 9 and 10, respectively, in Table 1). The arrowhead indicates the small delay in **c**.

**Figure 3:**
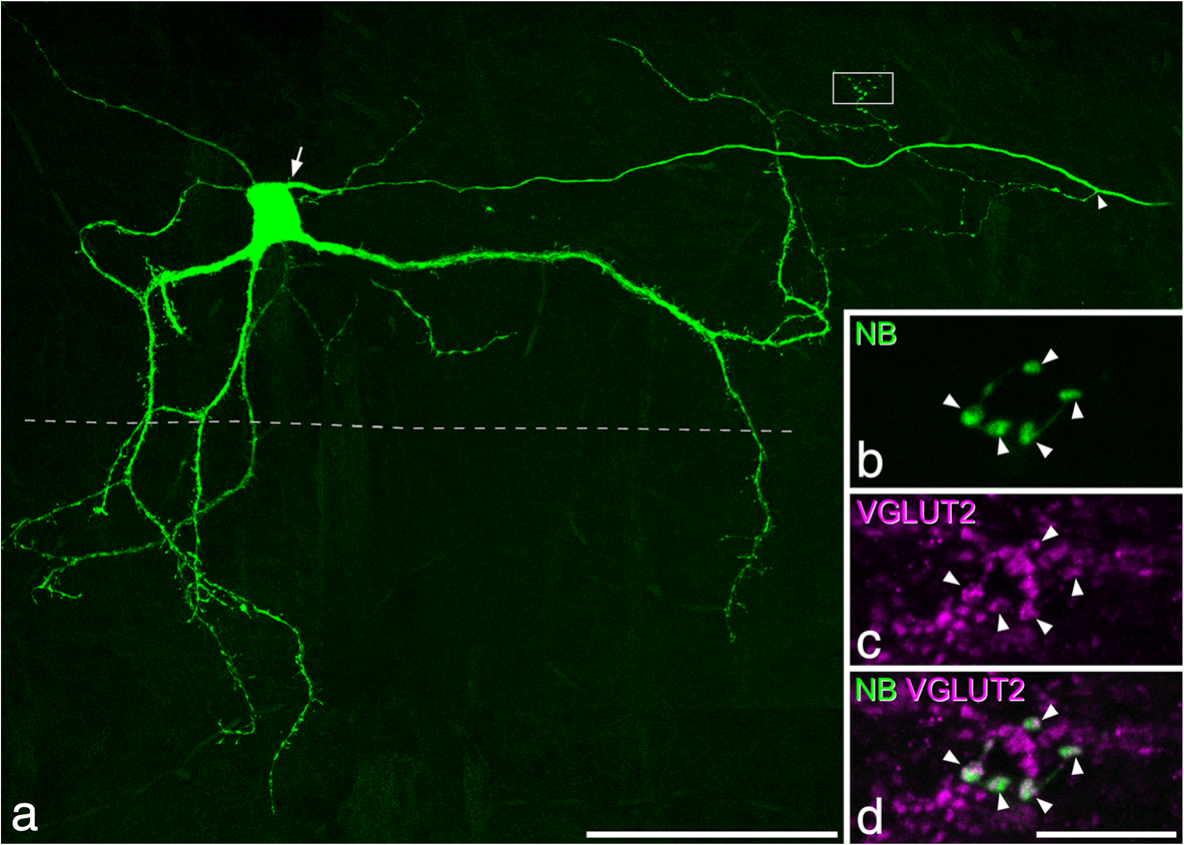
**VGLUT2 in the axon of one of the vertical cells from the CTb injected animals. a**, A projected confocal scan (51 z sections at 1 μm spacing) showing the morphology of one of the delayed-firing vertical cells (cell 10 in Table 1). The axon emerges from the right side of the soma (arrow) and gives off a single collateral (arrowhead), before leaving the slice. This collateral gives rise to branches with numerous boutons. The dashed line indicates the dorsal edge of the VGLUT1 plexus. **b**, A projection of 4 confocal optical sections (0.5 μm z-spacing) scanned to reveal Neurobiotin (NB) shows five boutons (arrowheads) belonging to the axon of this cell (some of those shown in the boxed area in **a**). **c**, The same field scanned to reveal VGLUT2. **d**, A merged image reveals that each of these boutons is VGLUT2-immunoreactive. Scale bars: 100 μm **(a)**, 10 μm **(b-d)**.

Three of the cells that were recorded in slices from the rats that had received injections of CTb into the ipsilateral sciatic nerve were classified morphologically as vertical cells. Two of these showed delayed firing in response to injection of depolarising current, while the other was defined as reluctant firing [6,41] (Figure 2). All three cells showed *I*_A_-like currents and one of them (cell 10 in Table 1) also showed *I*_h_- and *I*_Ca_-like currents. The axons of two of the cells could not be followed far enough to allow identification of boutons, but for one of the cells the axon was traced as far as the lamina I/II border, and we were able to identify 16 boutons that originated from it. These were all VGLUT2-immunoreactive (Figure 3), confirming that the cell was glutamatergic.

**Figure 4:**
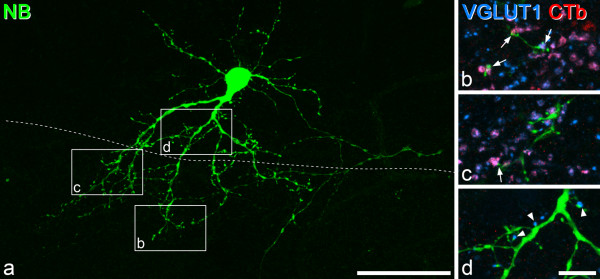
**CTb/VGLUT1 boutons contact vertical cell spines in a CTb injected animal. a**, Projection of a confocal image stack (75 optical sections at 0.5 μm z-spacing) to show one of the vertical cells from a rat that had received an injection of CTb into the ipsilateral sciatic nerve (cell 8 in Table 1). The dashed line shows the dorsal limit of the dense plexus of VGLUT1-immunoreactive axons, and the boxes show the positions of the images in the remaining parts of the figure. **b**, **c**, **d** show parts of the dendritic tree in fields scanned to reveal Neurobitin (green), VGLUT1 (blue) and CTb (red) in a stack of 7 optical sections (0.5 μm z-spacing). Arrows in **b** and **c** indicate contacts from VGLUT1^+^/CTb^+^ boutons onto dendritic spines of this cell. Arrowheads in **d** show contacts from small VGLUT1-immunoreactive boutons that lacked CTb onto dendrites in lamina IIo. Scale bars: 50 μm **(a)**, 10 μm **(b-d)**.

These cells (numbered 8–10 in Table 1) had between 97 and 283 dendritic spines that lay within the dense plexus of VGLUT1 axons, and 32–35% of these spines were in contact with at least one VGLUT1-immunoreactive bouton. In each case, many of the VGLUT1 boutons that contacted these spines were CTb-immunoreactive, and these accounted for 44–59% (mean 53%) of the VGLUT1 boutons in contact with the 3 cells. The great majority of the spines with VGLUT1 contacts received only a single contact, but in ~2% of cases two VGLUT1 boutons contacted the spine. Examples of contacts on one of these cells are illustrated in Figure 4. Although VGLUT1 boutons in laminae IIi-III that lacked CTb may have been primary afferents that were not anterogradely labelled, we observed some contacts from small VGLUT1^+^/CTb^-^ boutons onto dendritic spines of each of these 3 cells, just dorsal to the VGLUT1 plexus, an area that does not receive significant input from myelinated primary afferents that are transganglionically labelled with CTb [33,37,38,50-53] (Figure 4d).

## Discussion

The main findings of this study are that excitatory vertical cells in lamina II receive numerous contacts from VGLUT1-immunoreactive boutons on their dendritic spines in laminae IIi and III, and that many of these are primary afferents, as they are labelled with CTb following injection of the tracer into the sciatic nerve.

### Technical considerations

There is strong evidence that most vertical cells in lamina II are excitatory, since their axons express VGLUT2 [5,6] and paired recordings have shown that they generate EPSCs in their postsynaptic targets [17,18]. However, some lamina II neurons that closely resemble glutamatergic vertical cells express the vesicular GABA transporter VGAT, and are therefore inhibitory interneurons [5,6]. This indicates that morphology alone is not reliable for identifying excitatory vertical cells. We have found a clear difference in firing pattern between these two groups, since the glutamatergic vertical cells showed delayed or reluctant firing patterns, while the inhibitory cells fired tonically [6]. Delayed firing was also a consistent feature of vertical cells that were shown to be excitatory in paired recordings [17]. Although we could not demonstrate VGLUT2 in the axons of two of the cells recorded in the CTb-injected animals (cells 8 and 9 in Table 1), their firing patterns (delayed and reluctant) strongly suggest that these were glutamatergic.

We have previously shown that many VGLUT1-immunoreactive boutons in laminae IIi-III belong to myelinated primary afferents (A-LTMRs), as they can be labelled with CTb injected into a periperal nerve [33]. Consistent with this, Alvarez et al. [36] found that the majority of VGLUT1-immunoreactive boutons in this region were lost following multiple dorsal rhizotomies. We could not identify primary afferent-derived VGLUT1 boutons in the analysis of the first 7 vertical cells, because these were recorded in animals that had not received CTb injections. However, our findings in the 3 cells from the CTb-injected rats indicate that a significant proportion of the VGLUT1 boutons contacting the vertical cells are of primary afferent origin.

Without electron microscopy, it is not possible to confirm that the contacts we observed were synapses. However, dendritic spines are commonly associated with excitatory synapses and A-LTMRs often form the central boutons of synaptic glomeruli, which are presynaptic to several dendritic spines [54]. It is therefore likely that many of these contacts were associated with glutamatergic synapses. Although we saw numerous contacts between VGLUT1-immunoreactive boutons and the dendritic shafts of vertical cells, it is not clear whether these respresent synaptic contacts, because most of the postsynaptic structures in synaptic glomeruli are dendritic spines, rather than shafts [54]. For this reason, we did not quantify contacts onto dendritic shafts of the recorded neurons.

### Synaptic input to vertical cells

Electrophysiological studies in spinal cord slices have shown that vertical cells receive monosynaptic input from Aδ and C fibres [9,11,17], but could not reveal the receptive field properties of these afferents. It is not yet known which types of C fibre innervate these cells, but they are likely to include nociceptive afferents that express TRPV1, TRPA1 and/or Mas-related G protein-coupled receptor D (Mrgd) [15,16]. Bennett et al. [19] provided evidence that stalked cells in the cat are innervated by myelinated nociceptors, and the monosynaptic Aδ input to vertical cells is therefore likely to arise at least in part from Aδ nociceptors, which either terminate in lamina I/IIo or arborise diffusely throughout laminae I-V [55,56]. However, our results suggest that some of the monosynaptic Aδ input may originate from D-hair afferents, which express VGLUT1 [36] and project to laminae IIi-III [55,57,58], a region that is often penetrated by vertical cell dendrites. Gobel et al. [21] carried out an ultrastructural analysis of a lamina II stalked cell recorded *in vivo* in the cat, and reported that its dendritic spines received numerous synapses from the central axons of synaptic glomeruli. Interestingly, one of these central axons was particularly large, contained numerous mitochondria and had clusters of loosely-packed synaptic vesicles. This closely resembles the type II glomerular central endings identified in rat, which are thought to originate from Aδ D-hair afferents [54,59].

Aβ LTMRs, all of which express VGLUT1 [33,36], terminate throughout the deep dorsal horn (laminae III-VI), with some hair afferents and rapidly adapting afferents from glabrous skin penetrating into lamina IIi [57,58,60-63]. It is therefore possible that some of the contacts that vertical cell dendrites received from VGLUT1-immunoreactive boutons could represent synapses from Aβ LTMRs. Many studies have used electrical stimulation of dorsal roots to investigate primary afferent input to lamina II neurons in slice preparations, but very few of these neurons have been found to receive monosynaptic input from Aβ fibres [9,11,13,64-67]. However, this may be at least in part because of the difficulty of retaining these afferents intact in spinal cord slices. Aβ fibres have a complex projection, with collaterals arising from long branches that are orientated rostrocaudally in the dorsal columns. Many of these collaterals then pass through the dorsal horn or the medial part of the dorsal columns before curving laterally and entering laminae IIi-III from the deep aspect [58]. The preparation of the transverse, parasagittal or horizontal slices that were used in these studies, may therefore have disrupted the continuity between many Aβ LTMR boutons and their parent axons in the dorsal root, and could lead to failure to detect monosynaptic Aβ input. It is also possible that there were synapses between functionally intact Aβ LTMRs and vertical cells in these slice preparations, but that these were ineffective, either because they were silent [68,69], or because the resulting EPSCs were highly attenuated due to their distal location. One way to determine whether Aβ LTMRs synapse directly on vertical cells would be to record from these cells in slices from mice expressing green fluorescent protein (GFP) under control of the Npy2R promoter, in which central arborisations of Aβ afferents can be visualised directly through their expression of GFP [58].

Taken together, these observations suggest that vertical cells are innervated by a variety of different types of myelinated and unmyelinated primary afferents, including both nociceptors and LTMRs. Consistent with this interpretation, although some of the stalked cells recorded *in vivo* in the cat were nociceptive-specific, others had wide dynamic range receptive fields and responded to deflection of hairs [19].

Each of the vertical cells from animals that had received sciatic injections of CTb was contacted by boutons that were VGLUT1^+^/CTb^-^. While many of these could have belonged to myelinated afferents that had not taken up the injected tracer, some were located in lamina IIo, an area that contains virtually no labelled axons after CTb injection. Central terminals of unmyelinated primary afferents do not appear to contain detectable levels of VGLUT1 [33,36,44,70], but it is possible that these VGLUT1 boutons belong to a type of myelinated primary afferent that does not transport CTb (e.g. the nociceptors that terminate diffusely in laminae I-V [56]). An alternative explanation is that they are derived from corticospinal tract axons, which express VGLUT1 [34] and terminate in the superficial dorsal horn [71]. This raises the possibility that vertical cells are involved in the cortical modulation of pain pathways.

In addition to their primary afferent inputs, there is evidence that at least two classes of interneuron in lamina II are presynaptic to vertical cells. Lu and Perl [17] demonstrated excitatory inputs from transient central cells, while Zheng et al. [18] reported inhibitory inputs from cells expressing GFP under control of the Prion promoter (PrP-GFP cells).

### The role of vertical cells in sensory pathways

Gobel and colleagues were the first to suggest that stalked cells provided excitatory input to projection neurons in lamina I, since their axons can arborise extensively in this lamina [20,21]. Lu and Perl [17] provided direct support for this suggestion, by demonstrating monosynaptic excitatory connections from vertical cells to lamina I neurons, some of which were retrogradely labelled from rostral thoracic spinal cord. Further evidence was provided by Cordero-Erausquin et al. [72], who observed numerous vertical/stalked cells that were labelled with a method that allowed transfer of GFP to cells that were presynaptic to lamina I spinoparabrachial neurons. However, lamina I projection neurons are clearly not the only postsynaptic target for vertical cells, since their dendrites generally remain in this lamina [72-76], while the axons of vertical cells can arborise in laminae I, II and III. In addition, not all vertical cells have axons that can be followed into lamina I [6,9]. There is apparently no information about other potential targets, but these presumably include local interneurons, and possibly the dorsal dendrites of large ALT projection neurons in deeper laminae [77]. In addition to their fast synaptic actions, vertical cells may also give rise to slower, peptide-mediated, effects. We have reported that some vertical cells express somatostatin [6], which will act on the sst_2A_ receptors that are expressed by around half of the inhibitory interneurons in this region [3,10,78,79]. Interestingly, all of the PrP-GFP cells express sst_2A_[80], and somatostatin released from vertical cells may therefore suppress their inhibitory input from this class of interneuron [18].

Some forms of tactile allodynia in neuropathic pain are evoked by activation of myelinated LTMRs [81,82], and it is thought that loss of inhibition in the dorsal horn is an important contributor to neuropathic pain [83-86], although there is debate about the underlying mechanisms [4,65,87-91]. Torsney and McDermott [31] proposed that disinhibition could open up a polysynaptic pathway that connected myelinated LTMRs to lamina I neurons, leading to allodynia as a result of increased low-threshold drive to projection cells that are normally activated mainly by nociceptive inputs. They represented this diagramatically as a chain of excitatory interneurons that extended dorsally from lamina III, where most A-LTMR afferents terminate, because there is little evidence for lamina III interneurons with significant axonal projections to lamina I [92]. Consistent with this suggestion, Lu et al. [32] have recently provided evidence for a polysynaptic pathway involving PKCγ-expressing excitatory interneurons in laminae IIi-III [93] that are directly innervated by Aβ afferents and activate a class of excitatory interneuron in lamina II (transient central cells), which in turn excite vertical cells. They proposed that this circuit was under feed-forward inhibition from glycinergic neurons in lamina III, and that following spinal nerve ligation [94], the inhibition was reduced, leading to strengthening of polysynaptic Aβ pathways [32]. However, it is not clear whether this would contribute to tactile allodynia, since these changes were observed in the L5 segment (which had input from damaged primary afferents), but not following stimulation of the L4 root, which is thought to be responsible for conveying inputs that give rise to allodynia in this model [95].

**Figure 5:**
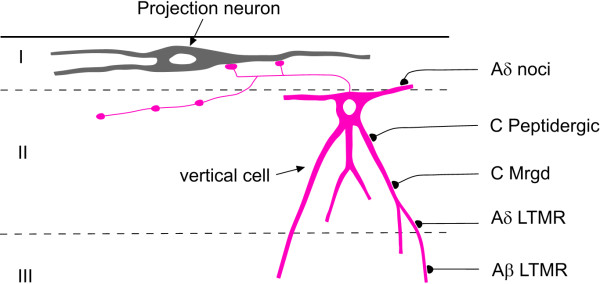
**Proposed disynaptic circuit linking myelinated low-threshold primary afferents and lamina I projection neurons.** The present results, together with findings from previous studies, suggest that vertical cells in lamina II receive monosynaptic input from a variety of types of primary afferent, including Aδ nociceptors (noci), both peptidergic and non-peptidergic (Mrgd-expressing) C fibres, as well as Aδ and/or Aβ low threshold mechanoreceptors (LTMR). Their postsynaptic targets include projection neurons in lamina I. For convenience, dendritic spines, which are likely to be major sites of excitatory synapses on vertical cells, have been omitted.

Although the polysynaptic pathway described above presumably can convey A-LTMR input to lamina I projection neurons, our results suggest that there may be an additional, more direct route, as shown in Figure 5. This would involve monosynaptic input from A-LTMRs to the ventral dendrites of vertical cells, thus providing a disynaptic link between these afferents and lamina I projection neurons. As stated above, some vertical cells have axons that do not enter lamina I, and therefore these cells presumably cannot innervate lamina I projection neurons. However, our finding that all of the vertical cells with dendrites that entered laminae IIi-III received numerous contacts from VGLUT1 boutons, suggests that the vertical cells that are presynaptic to lamina I projection neurons are likely to receive low-threshold input. In normal conditions, this distal input could be insufficient to cause the vertical cells to fire, possibly because the A-LTMR afferents are powerfully inhibited by local inhibitory neurons that form axo-axonic synapses with their central terminals [96]. However, in a disinhibited state, these afferents may be capable of eliciting action potentials in vertical cells, which would then excite lamina I projection neurons, leading to mis-coding of tactile inputs as nociceptive.

## Conclusions

The present results suggest that in addition to their nociceptive input, lamina II vertical cells may receive synapses from myelinated low-threshold mechanoreceptors on their ventral dendrites. Vertical cells could therefore sample a diverse range of sensory input, and serve to integrate this before transmitting it to projection neurons in lamina I. Strengthening of this putative disynaptic pathway between tactile afferents and projection cells could contribute to the allodynia seen in neuropathic pain.

## Abbreviations

ALT: Anterolateral tract; CTb: Cholera toxin B subunit; GFP: Green fluorescent protein; IA: A-type potassium current; ICa: Calcium current; Ih: Hyperpolarisation-activated current; LPb: Lateral parabrachial area; LTMR: Low-threshold mechanoreceptor; NA: Numerical aperture; VGLUT1: Vesicular glutamate transporter 1; VGLUT2: Vesicular glutamate transporter 2.

## Competing interests

The authors declare that they have no competing interests.

## Authors’ contributions

TY, JSR, EK and AJT participated in the design of the study; TY performed the patch-clamp experiments and analysed the resulting data; SYXT and EP analysed the anatomical data; MW generated antibodies used in the study. TY, SYXT, EP, MW, JSR and AJT contributed to the writing of the manuscript and all authors approved the final version.
